# Epidemiological characteristics of asthma-COPD overlap, its association with all-cause mortality, and the mediating role of depressive symptoms: evidence from NHANES 2005–2018

**DOI:** 10.1186/s12889-024-18911-1

**Published:** 2024-05-28

**Authors:** Meng Zhu, An Chen

**Affiliations:** 1 School of Public Health, Zhejiang Chinese Medical University, No. 548 Binwen Road, Binjiang District, Hangzhou, Zhejiang Province 310053 China; 2 School of Basic Medicine, Zhejiang Chinese Medical University, No. 548 Binwen Road, Binjiang District, Hangzhou, Zhejiang Province 310053 China; 3 Department of Public Health, Faculty of Medicine, University of Helsinki, Biomedicum 1, Helsinki, 00290 Finland

**Keywords:** Asthma-COPD overlap, Depressive symptoms, All-cause mortality, Mediation analysis

## Abstract

**Background:**

Asthma-COPD overlap (ACO) is a distinct and intricate respiratory condition that requires specific attention and management. The objective of this cohort study was to examine the epidemiological characteristics of ACO, explore the association between ACO and all-cause mortality, and investigate the potential mediating role of depressive symptoms in this association.

**Methods:**

This retrospective cohort study used data from the National Health and Nutrition Examination Survey (NHANES) 2005–2018 and National Death Index (NDI) 2019. A total of 22,745 participants were included: 705 with ACO, 2352 with asthma-only, 853 with COPD-only, and 18,835 without asthma or COPD. The non-ACO group (*N* = 22,040) referred to the individuals without ACO. Statistical tests were employed to assess differences in some characteristics between the ACO group and the other groups. Cox proportional hazards models were applied to evaluate the relationship between ACO and all-cause mortality, estimating hazard ratios (HR) with 95% confidence intervals. Mediation analysis was conducted to investigate the potential mediating effects of depressive symptoms on the association of ACO with all-cause mortality.

**Results:**

The prevalence of ACO was 3.10% in our study population. Compared to the non-ACO participants, the ACO participants exhibited significantly different characteristics, including higher age, a lower family income-to-poverty ratio, a higher body mass index, higher rates of comorbidities i.e., hypertension, diabetes, hyperlipidemia, cardiovascular disease, and cancer, poorer dietary habits, and a higher rate of depressive disorders. Compared to the participants without ACO, the participants with ACO exhibited a significant increase in all-cause mortality (HR = 1.908, 95%CI 1.578–1.307, *p* < 0.001). The proportions mediated by depressive symptoms for ACO -associated all-cause mortality were 8.13% (CI: 4.22%-14.00%, *p* < 0.001).

**Conclusions:**

This study revealed a strong relationship between ACO and all-cause mortality and uncovered a potential psychological mechanism underlying this relationship. Our study indicates the possible necessity of offering comprehensive care to ACO patients, encompassing early detection, lifestyle guidance, and mental health support. Nevertheless, due to the limitations in the study design and the dataset, the results should be interpreted with caution.

**Supplementary Information:**

The online version contains supplementary material available at 10.1186/s12889-024-18911-1.

## Background

The two common progressive lung diseases, asthma and chronic obstructive pulmonary disease (COPD), are leading causes of morbidity and mortality worldwide [1]. They have unique clinical features requiring different management strategies but can coexist. Despite a lack of consensus on its recognition, definition, and diagnosis [2, 3], the term "Asthma-COPD overlap" (ACO), describing a condition where individuals show symptoms of both asthma and COPD, has gained widespread use since it was recommended by the Global Initiative for Asthma (GINA) and the Global Initiative for Chronic Obstructive Lung Disease (GOLD) in 2017 [2, 4, 5]. While the debate about the term is still ongoing, there is agreement among healthcare professionals that patients presenting symptoms of both Asthma and COPD require personalized care for better outcomes [3, 6]. Despite the recommendation by the GOLD report in 2021 to discontinue the term "ACO" [7], ACO as a medical condition or trait remains a subject of considerable interest and discussion, attracting significant attention [8]. As proposed by various recommendations and guidelines, ACO can be broadly understood as a medical condition characterized by persistent airflow limitation and clinical features that exhibit characteristics of both asthma and COPD [6, 8].

Due to a lack of consensus on identification or diagnostic criteria, the prevalence of ACO has not been firmly established [3]. However, currently available evidence suggests that ACO is not uncommon, with a prevalence ranging from 1 to 30% in the general population, representing around 10%-60% of COPD patients and around 15%-66% of asthma patients [2, 3, 6, 8–10]. It has been estimated that the number of ACO patients will increase significantly alongside the rising numbers of asthma and COPD patients [6]. Patients with ACO may experience a greater symptom burden, worse quality of life and more frequent and severe respiratory exacerbations than those with asthma or COPD alone, resulting in increased healthcare services and substantial economic costs [6, 8, 10]. Therefore, there is an urgent need to develop a comprehensive understanding of ACO and implement appropriate measures to address it effectively.

Substantial progress has been made in ACO research [8], but current knowledge and experience for ACO and its management are notably limited [11]**.** Our current understandings of ACO and the targeted treatments and care are mainly informed by studies in asthma and COPD that usually excluded ACO patients [2, 6, 8]. From an epidemiological perspective, characteristics of ACO have not been thoroughly studied and current evidence holds conspicuous inconsistencies. While Inoue et al. (2017) [12] found patients identified as having ACO syndromes were significantly younger, Koleade et al. (2018) and Ekerljung et al. (2018) suggested a higher age was associated with an increased risk of ACO [13, 14]. Lee et al. (2021) and Koleade et al. (2018) presented conflicting conclusions regarding the association between gender and ACO [13, 15]. Although many studies have reported that ACO is associated with increased mortality [16, 17], Leung and Sin (2023) [8] have suggested that the current evidence about the mortality related to ACO is still limited and even inconsistent, partially attributed to the varying criteria of identification and different populations.

While mental health is crucial in managing physical health issues and improving the quality of patients’ lives [18], research on mental health conditions like depression among ACO patients is scarce. Depression, which has a strong association with higher mortality rates [19] and has gained significant attention globally in recent years, was widely reported as a prevalent comorbidity among patients with lung diseases, including asthma and COPD [20–22]. Numerous studies have investigated how depression may mediate the relationship between lung diseases and mortality or adverse health outcomes, aiming to develop targeted interventions for managing these diseases and preventing negative consequences [22]. However, current understandings of the association between ACO and depression are limited to specific observations in asthma and COPD patients [23, 24], and the role of depression in the development of ACO remains unclear.

This study aims to advance current understandings of ACO by investigating 1) the prevalence and epidemiological characteristics of ACO using the commonly used definition of ACO [25] and well-accepted approaches to its identification, 2) the association of ACO with all-cause mortality, and 3) the potential mediating role of depressive symptoms in this association.

## Methods

### Study design and population

We conducted a retrospective longitudinal study utilizing the National Health and Nutrition Examination Survey (NHANES) 2005–2018 database, and National Death Index (NDI) records that were updated to December 31, 2019. NHANES is a national cross-sectional survey program conducted by the National Center for Health Statistics (NCHS) of the Centers for Disease Control and Prevention (CDC) in two-year cycles, with a goal of providing insights into various health conditions, risk factors, and nutritional patterns of families and populations in the United States (https://www.cdc.gov/nchs/nhanes/index.htm). NHANES offers a distinctive opportunity for studying ACO due to its provision of a representative sample of approximately 5000 individuals on a yearly basis, combining data from interviews, physical examinations, and laboratory tests (datasets from every two years are incorporated into one cycle) [1, 26]. The dataset encompasses Demographic Data, Dietary Data, Examination Data, Laboratory Data, Questionnaire Data, and Limited Access Data.

The NHANES data can be linked with death certificate records from the National Death Index (NDI), i.e., Linked Mortality Files (LMFs) provided by NCHS, which have been updated with mortality follow-up data through December 31, 2019. For protecting the identities of the individuals involved, the NHANES database anonymizes participants’ data and employs unique identifiers (called ‘SEQN’) that could help match participants’ data with LMFs. For more information on accessing the mortality files, please refer to the official website (https://www.cdc.gov/nchs/data-linkage/mortality.htm) [27].

For this investigation, we retrieved data from seven NHANES cycles spanning the years from 2005 to 2018, specifically encompassing the 2005–2006, 2007–2008, 2009–2010, 2011–2012, 2013–2014, 2015–2016, and 2017–2018 cycles, and a total of 70,190 individuals were identified. After excluding the ones with missing information on depressive symptoms assessed by the PHQ-9 questionnaire (*N* = 33,800), pulmonary disease diagnosis (*N* = 2,095), and mortality (*N* = 64), 34,231 individuals were selected. Since one well-accepted criterion in identifying both ACO and COPD was an age equal to or over 40 [28–30], in this study we excluded 11,486 individuals whose age was less than 40 years. Finally, 22,745 participants were included in the analysis. NHANES created weights applied to data to account for oversampling, nonresponse, and noncoverage, thereby forming representative samples of the U.S. civilian noninstitutionalized resident population. In this study involving Mobile Examination Center (MEC) data, the 2-year sample weight– wtmec2yr accounted for 7 cycles (1/7*wtmec2yr) was used for all analyses, which allowed for the generation of nationally representative estimates with a weighted population estimated to be around 130 million. More information about the weights and their associated process could be found on the NHANES website via the following link: https://wwwn.cdc.gov/nchs/nhanes/tutorials/weighting.aspx.

### ACO and the referenced groups

**Fig. 1:**
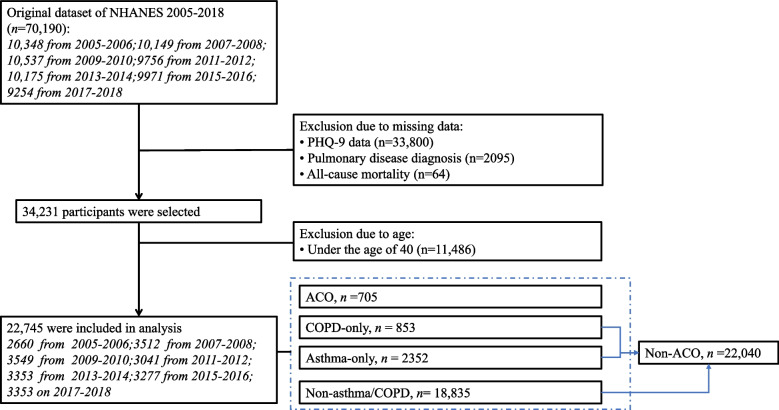
The flow chart of identifying study population and grouping

By using the commonly used criteria of or available approaches to identifying the focused diseases and clinical conditions, and relying on the data availability within the NHANES 2005–2018, this study grouped participants as follows: 1) the asthma-only group (*N* = 2352) [1, 4, 31–33], including participants who reported at least one of the following conditions: a) having an episode of asthma or an asthma attack during the past 12 months, b) ever being told by a doctor or other health professionals that they had asthma, and c) using drugs including selective phosphodiesterase-4 inhibitors, mast cell stabilisers, leukotriene modifiers or inhaled corticosteroids, and excluding participants with chronic bronchitis and emphysema; 2) the COPD-only group (*N* = 853) [4, 31, 34, 35], including participants who had at least one of the following conditions: a) reporting having emphysema, b) reporting ever being told they had emphysema, c) having a ratio of forced expiratory volume in 1 s (FEV1) and forceful lung volume (FVC) (FEV1/FVC) after bronchodilator use less than 0.70 in laboratory test, and d) reporting ever being told they had chronic bronchitis and used drugs including selective phosphodiesterase-4 inhibitors, mast cell stabilisers, leukotriene modifiers, inhaled corticosteroids, and excluding participants with asthma; 3) the ACO group (*N* = 705) [4, 6, 36, 37], including participants who met the identification criteria for both asthma and COPD (at least 1 characteristic from each); 4) the non-asthma/COPD group, including participants who did not meet any identification criteria for the asthma-only or COPD-only group. We formed the non-ACO group by combining participants from the asthma-only, COPD-only, and non-asthma/COPD groups. In this study, smoking was not considered as a condition to identify COPD [38, 39], mainly because while it has been regarded as the best-known and most important risk factor for irreversible airflow obstruction, non-smokers can also develop COPD [40]. Figure 1 is the flow chart of identifying study population and grouping.

### All-cause mortality as the outcome

This study defined the outcome as all-cause mortality, i.e., death due to any cause, using the data extracted from NHANES linked Public-Use Linked Mortality Files through December 31, 2019. Participants with mortality status 0 (MORTSTAT = 0) were considered to be alive through the end of 2019. The follow-up period started at baseline defined as the date of NHANES participation and ended on the date of death or December 31, 2019 if the participant was still alive.

### Depressive symptoms

Rather than a diagnostic tool, the Patient Health Questionnaire-9 (PHQ-9), a nine-item instrument that utilizes a 0–3-point Likert scale containing 9 items totaling 27 points, is widely recognized as a brief, reliable and valid screening or assessment tool for depression [41, 42]. Since 2005, the NHANES program has utilized the PHQ-9 to evaluate participants' depressive symptoms, and the majority of depression-related studies using the NHANES database have employed the PHQ-9 as the measurement for depression, with a PHQ-9 score of ≥ 10 indicating major depression [43, 44]. In this study, the assessment of participants' depressive status was conducted using PHQ-9 scores, and a score of 10 was set as the cut-off point indicating the presence of depressive disorders or clinically relevant depression (CRD) [42, 45, 46].

## Covariates

Variables with available data from the NHANES 2005–2018 (missing data < 60%), representing the baseline characteristics of the participants, and identified as potential confounding factors that could exert an influence on the outcome based on previous studies, were considered in this study and grouped into three categories as follows:

1) demographic characteristics, including age (years), gender (male, female), race (non-Hispanic White, non-Hispanic Black, Mexican American, other races), marital status (married or living with partner, widowed/divorced/separated, never married), education (less than high school, high school or equivalent, college and above), and family income to poverty ratio (PIR) (a proxy measure for socio-economic status, calculated by dividing household or individual income by a poverty threshold specific to the survey year and state) [47–52]; 2) other medical conditions, including body mass index (BMI), hypertension (no/yes) (defined by systolic blood pressure ≥ 140 mmHg, diastolic blood pressure ≥ 90 mmHg, taking blood pressure medication, or participants self-reporting having hypertension), diabetes mellitus (DM) (no/yes) (defined by participants’ self-report of having diabetes mellitus or having a previous diagnosis of diabetes mellitus), hyperlipidemia (no/yes) (defined by participants’ self-report of using cholesterol-lowering medications, triglycerides ≥ 150 mg/dL, total cholesterol ≥ 200 mg/dL, low-density lipoprotein ≥ 130 mg/dL, or HDL ≤ 40 mg/dL in males and ≤ 50 mg/dL in females), cancer (no/yes) (defined by participants’ self-report of being told by a doctor or another health professional that they had cancer or a malignancy of any kind), and cardiovascular disease (CVD) (no/yes) (defined if the respondents reported being told by a doctor or another health professional that they had a diagnosis of congestive heart failure, coronary heart disease, angina, heart attack, or stroke) [53–55]; 3) health-related lifestyle, including alcohol intake ≥ 12 drinks per year (no/yes) (defined by participants’ self-report of having at least 12 drinks of any type of alcoholic beverage in one year), healthy eating index (HEI) (0–100, a higher score indicating better adherence to the 2010–2015 Dietary Guidelines for Americans) [56–58], and serum cotinine level (ng/ml), a biomarker of the exposure to tobacco smoke and reflecting smoking status (both active and passive), with a higher level indicating greater exposure to tobacco smoke [38, 59, 60].

### Statistical analyses

Our analysis incorporated the complex sampling design and considered the sampling weight -wtmec2yr/7 to enhance representativeness and accuracy in estimation. The normality of the distribution of continuous variables in the dataset was assessed by Kolmogorov–Smirnov test, and tests showed that all continuous variables included in this study satisfied or almost satisfied with the assumption of normality. Descriptive statistics included mean and standard deviation for continuous data, and frequencies and proportions for categorical data. Student t-tests were used to estimate if there were significant differences between the ACO participants and the ones from other groups (asthma-only, COPD-only, non-Asthma/COPD, or non-ACO) in terms of characteristics measured by continuous variables, and the Chi-square (χ2) tests used for characteristics measured by categorical variables.

Weighted univariable and multivariable Cox proportional hazards models were employed to perform survival analyses, examining the relationship between ACO (exposure) and all-cause mortality (event). The hazard ratio (HR) with 95% confidence intervals (CIs) was estimated. The follow-up period commenced at the time of the individual’s participation in the NHANES program and concluded on the date of death or December 31, 2019. Before conducting the survival analyses, we evaluated the proportional hazards assumption using the Schoenfeld individual test, with a significance level of *p* < 0.05 indicating a violation, and observed that the assumption was met (see Additional file 1). In the modelling process, we intentionally introduced the groups of covariates step by step, gradually controlling for potential confounders representing demographic characteristics, other medical conditions, and health-related lifestyle, to observe the specific effects of different confounding factors on the outcome and the changes in the model’s explanatory power.

In detecting the potential mediating role of depressive symptoms in the association between ACO and all-cause mortality, we applied the strategy of distribution–of–the–product. The assessment of the mediating role of depressive symptoms between ACO (non-ACO, i.e., the combination of asthma-only, COPD-only, and non-asthma/COPD as the reference) and all-cause death involved the following steps: 1) examining the total effect of ACO on all-cause mortality after controlling for all covariates, 2) analyzing the direct effect of ACO on all-cause mortality after controlling for all covariates and depressive symptoms, 3) evaluating the indirect effect of ACO on all-cause mortality through depressive symptoms after controlling for all covariates, and 4) determining the proportion mediated by depressive symptoms in the link between ACO and all-cause mortality (i.e., indirect effect/total effect). In order to capture more nuanced variations in the relationship between the variables and to establish a more precise understanding of the underlying mechanisms and the strength of the mediation effect, we treated the hypothesized mediator, depressive symptoms, as a continuous variable in the present mediation analysis.

Statistical analyses were conducted using R version 4.3.0 (https://www.r-project.org/) and, when applicable, SPSS 26. The mediation analysis was performed using the available R package named “mediation” [61]. Two sided *p*-value < 0.05 was considered statistically significant.

### Ethical approval

The NCHS ethical review board approved all NHANES protocols, and written informed consent was obtained from all participants.

### Data sharing

The data from NHANES and NDI are publicly available.

## Results

### Characteristics of study population

The selected characteristics of our participants are showed in Table 1. Among the total of 22,745 individuals aged ≥ 40 years old, there are 705 participants with ACO, 2352 with asthma-only, 853 with COPD-only, and 18,835 without asthma or COPD. The prevalence of ACO was 3.10% in our study population, 23.08% among the participants having asthma (the ACO group / [the ACO group + the asthma-only group]), and 45.25% having COPD (the ACO group / [the ACO group + the COPD-only group]).

**Table 1:** Characteristics of study population (*N* = 22,745)

Characteristics	Total, *N* = 22,745	ACO, *N* = 705	Asthma-only, *N* = 2352	COPD-only, *N* = 853	Non-asthma/COPD, *N* = 18,835	Non-ACO, *N* = 22,040	*p*-value (ACO vs Asthma-only)	*p*-value (ACO vs COPD-only)	*p*-value (ACO vs Non-asthma/COPD)	*p* -value (ACO vs Non-ACO) h
**Age**^a^	57.84 ± 0.17	60.80 ± 0.50	56.35 ± 0.31	62.49 ± 0.42	57.71 ± 0.18	57.75 ± 0.17	t = 9.127, < 0.001^e^	t = -4.547, 0.01^e^	t = 6.026, < 0.001^e^	t = 6.008, < 0.001^e^
**Gender**^b^							χ2 = 12.831, 0.66 ^d^	χ2 = 62.656, < 0.001 ^d^	χ2 = 4.799, 0.003 ^d^	χ2 = 3.314, 0.01^d^
Female	11,573(52.46)	383(60.79)	1457(62.12)	292(40.16)	9441(51.48)	11,190(52.20)				
Male	11,172(47.54)	322(39.21)	895(37.88)	561(59.84)	9394(48.52)	10,850(47.80)				
**Race**^b^							χ2 = 40.892, 0.02 ^d^	χ2 = 17.548, 0.02 ^d^	χ2 = 76.959, < 0.001 ^d^	χ2 = 66.112, < 0.001 ^d^
Non-Hispanic White	10,228(72.83)	405(77.38)	1040(72.50)	575(84.57)	8208(72.17)	9823(72.69)				
Non-Hispanic Black	4984(10.25)	146(8.97)	624(12.85)	138(5.97)	4061(10.14)	4823(10.28)				
Mexican American	3241(6.22)	40(2.16)	230(4.13)	44(1.61)	2927(6.84)	3201(6.34)				
Other races	4307(10.72)	114(11.49)	458(10.52)	96(7.86)	3639(10.84)	4193(10.69)				
**Marital**^b^							χ2 = 14.843, 0.01 ^d^	χ2 = 10.263, 0.71 ^d^	χ2 = 45.809, < 0.001 ^d^	χ2 = 40.442, < 0.001 ^d^
Never married	1830(6.98)	58(5.99)	225(8.26)	49(4.12)	1498(6.98)	1772(7.01)				
Married	14,061(67.59)	361(61.35)	1350(64.35)	502(64.71)	11,848(68.41)	13,700(67.82)				
Widowed/Divorced/Separated	6839(25.43)	286(32.66)	774(27.39)	302(31.17)	5477(24.61)	6580(25.2)				
**Education**^b^							χ2 = 11.536, 0.01 ^d^	χ2 = 10.165, 0.01 ^d^	χ2 = 1.974, 0.14 ^d^	χ2 = 2.176, 0.11^d^
Below high school	2848(5.93)	95(8.05)	231(4.57)	91(5.84)	2431(6.03)	2753(5.87)				
High school or equivalent	8500(34.25)	275(34.79)	872(33.40)	395(43.86)	6958(33.92)	8225(34.25)				
College or above	11,374(59.77)	334(57.15)	1248(62.03)	367(50.30)	9425(60.05)	11,040(59.88)				
**PIR**^a^	3.21 ± 0.04	2.64 ± 0.10	3.14 ± 0.07	2.97 ± 0.10	3.25 ± 0.04	3.22 ± 0.04	t = -4.618, < 0.001^e^	t = -3.241, 0.01^e^	t = -7.625, < 0.001^e^	t = -7.235, < 0.001^e^
**BMI**^a^	29.51 ± 0.08	30.96 ± 0.48	31.22 ± 0.25	28.55 ± 0.31	29.28 ± 0.08	29.46 ± 0.08	t = -1.627, < 0.001^e^	t = 7.287, 0.01^e^	t = 6.826, < 0.001^e^	t = 5.853, 0.003^e^
**Hypertension**^b^							χ2 = 10.726, 0.01 ^d^	χ2 = 10.581, 0.01 ^d^	χ2 = 57.632, < 0.001 ^d^	χ2 = 49.965, < 0.001 ^d^
No	9820(48.74)	213(35.34)	871(44.04)	326(43.89)	8410(50.06)	9607(49.16)				
Yes	12,922(51.25)	492(64.66)	1481(55.96)	527(56.11)	10,422(49.94)	12,430(50.84)				
**DM**^b^							χ2 = 4.456, 0.02 ^d^	χ2 = 10.806, 0.05 ^d^	χ2 = 44.634, < 0.001 ^d^	χ2 = 37.227, < 0.001 ^d^
No	18,189(84.70)	501(76.79)	1757(81.54)	669(82.45)	15,262(85.59)	17,688(85.02)				
Yes	4538(15.21)	204(23.21)	593(18.46)	184(17.55)	3557(14.41)	4334(14.98)				
**Hyperlipidemia**^b^							χ2 = 3.704, 0.08 ^d^	χ2 = 3.696, 0.66 ^d^	χ2 = 8.960, 0.02 ^d^	χ2 = 8.405, 0.02^d^
No	5081(21.70)	126(17.08)	501(21.06)	187(18.20)	4267(22.11)	4955(21.84)				
Yes	17,663(78.30)	579(82.92)	1851(78.94)	666(81.80)	14,567(77.89)	17,084(78.16)				
**CVD**^b^							χ2 = 77.668, < 0.001 ^d^	χ2 = 8.589, 0.001 ^d^	χ2 = 258.732, < 0.001 ^d^	χ2 = 220.029, < 0.001 ^d^
No	19,101(86.89)	450(66.57)	1882(84.29)	605(76.11)	16,164(88.46)	18,651(87.52)				
Yes	3641(13.11)	255(33.43)	470(15.71)	248(23.89)	2668(11.54)	3386(12.48)				
**Cancer**^b^							χ2 = 18.775, < 0.001 ^d^	χ2 = ,1.518 0.91 ^d^	χ2 = 41.809, < 0.001 ^d^	χ2 = 36.773, < 0.001 ^d^
No	19,631(85.07)	554(75.90)	2009(84.34)	681(76.21)	16,387(86.01)	19,077(85.45)				
Yes	3092(14.82)	150(24.10)	340(15.66)	172(23.79)	2430(13.99)	2942(14.55)				
**HEI**^a^	51.83 ± 0.24	49.43 ± 0.70	50.62 ± 0.48	49.84 ± 0.79	52.16 ± 0.24	51.90 ± 0.24	t = -2.474, < 0.001^e^	t = -0.897, < 0.001^e^	t = -4.637, < 0.001^e^	t = -4.229, < 0.001^e^
**Alcohol drinks ≥ 12 per year**^b^							χ2 = 6.613, 0.02 ^d^	χ2 = 4.382, 0.47 ^d^	χ2 = 10.402, 0.01 ^d^	χ2 = 10.152, 0.01^d^
No	7859(27.17)	268(34.57)	810(28.16)	301(32.32)	6480(27.75)	7591(27.97)				
Yes	13,960(69.30)	397(65.43)	1442(71.84)	518(67.68)	11,603(72.25)	13,563(72.03)				
**Serum cotinine(ng/ml)**^a^	0.057 ± 0.001	0.092 ± 0.007	0.057 ± 0.004	0.122 ± 0.008	0.053 ± 0.018	0.056 ± 0.002	t = 8.582, < 0.001^e^	t = -2.493, < 0.001^e^	t = 9.818, < 0.001^e^	t = 9.162, < 0.001^e^
**Depressive disorders**^b^							χ2 = 31.051, < 0.001 ^d^	χ2 = 31.553, < 0.001 ^d^	χ2 = 214.207, < 0.001 ^d^	χ2 = 303.69, < 0.001 ^d^
No	20,636(92.08)	539(80.97)	2008(87.36)	745(89.86)	17,344(93.19)	20,097(92.42)				
Yes^f^	2109(7.92)	166(19.03)	344(12.64)	108(10.14)	1491(6.81)	1943(7.58)				
**All-cause mortality**^b^							χ2 = 181.550, < 0.001 ^d^	χ2 = ,0.126 0.58 ^d^	χ2 = 169.765, < 0.001 ^d^	χ2 = 4161.158, < 0.001 ^d^
No	19,283(88.65)	478(72.90)	2094(92.36)	570(74.55)	16,141(89.39)	18,805(89.13)				
Yes	3462(11.35)	227(27.10)	258(7.64)	283(25.45)	2694(10.61)	3235(10.87)				
**Follow-up time(months)**^a,g^	86.63 ± 1.14	78.70 ± 2.67	85.86 ± 1.80	91.54 ± 2.17	86.79 ± 1.16	86.88 ± 1.14	t = 3.467, 0.002^e^	t = -4.032, < 0.001^e^	t = -5.231, 0.003^e^	t = -5.099, < 0.001^e^

Legends: The selected characteristics of the participants are showed in Table 1

*Abbreviations*: *ACO* Asthma-chronic obstructive pulmonary disease overlap, *COPD* Chronic Obstructive Pulmonary Disease, *PIR* family poverty income ratio, *BMI* body mass index, *HEI* healthy eating index, *DM* diabetes mellitus, *CVD* cardiovascular disease, *CRD* clinically relevant depression

^a^Mean ± SE

^b^n, numbers of subjects; %, weighted percentage

^c^One-way ANOVA

^d^Pearson's Chi-squared test

^e^ Welch Two Sample t-test

^f^In this study those with PHQ-9 total scores ≥ 10 were considered as having depressive disorders or clinically relevant depression (CRD)

^g^Follow-up duration between the individual’s participation to NHANES program and December 31, 2019

^h^Two groups (the ACO group and the non-ACO group) were compared

Compared to those from the non-ACO group (combination of the asthma-only, COPD-only, and non-asthma/COPD groups), participants from the ACO group were more likely to be older, had a higher rate of reporting races of non-Hispanic white and other races, had a higher rate of having unsatisfied marriage outcomes (widowed/divorced/separated), had a lower PIR, had a higher BMI, had higher rates of suffering other diseases, including hypertension, DM, hyperlipidemia, CVD, and cancer, had a lower HEI, had a higher level of serum cotinine, consumed less alcohol, and were more likely to suffer depressive disorders (*p* < 0.05 for all comparisons). Compared to those with asthma-only, COPD-only or non-asthma/COPD respectively, participants with ACO had a higher rate of reporting races rather than non-Hispanic White, non-Hispanic Black, or Mexican American, a lower PIR, higher rates of suffering from hypertension, DM, and CVD, a lower HEI, and a higher rate of suffering from depressive disorders (*p* < 0.05 for all comparisons).

In addition, in comparison to the non-asthma/COPD group, besides the differences in the characteristics mentioned above, the ACO group exhibited significantly higher age, a higher proportion of females, a higher percentage of unmarried participants, an elevated BMI, a lower prevalence of hyperlipidemia, but a lower proportion of consuming alcohol at a rate of ≥ 12 drinks per year (*p* < 0.05 for all comparisons). Compared to the asthma-only group, the ACO group had more participants with widowed/divorced/separated, a lower level of education, a higher rate of cancer, and a lower BMI (*p* < 0.05 for all comparisons). Compared to the COPD-only group, the ACO group had more female and more participants with an education level of below high school and college or above, but experienced a lower level of serum cotinine (*p* < 0.05 for all comparisons).

### Association between ACO and all-cause mortality

Table 1 shows the survival status of different groups. During an average of 86.63-month follow-up (from the time point of the individual’s participantion to the NHANES program to the date of death or December 31, 2019), 3462 all-cause deaths occurred, accounting for 11.35% of the total study population, including 227 (27.10%) from the ACO group, 258(7.64%) from the asthma-only group, 283(25.45%) from the COPD-only group, and 2694(10.61%) from the non-asthma/COPD group. According to Table 1, compared to those from the non-ACO group, participants from the ACO group had a higher rate of all-cause death (*p* < 0.001). More specifically, compared to the asthma-only group and the non-asthma/COPD group respectively, the ACO group had significantly higher rates of all-cause mortality (*p*-value of ACO vs asthma-only < 0.001, *p*-value of ACO vs non-asthma/COPD < 0.001). Meanwhile, the ACO group has a significantly shorter period of follow-up (*p*-value of ACO vs asthma-only = 0.002, *p*-value of ACO vs COPD-only < 0.001, and *p*-value of ACO vs non-asthma/COPD = 0.003).

Table 2 shows the results of weighted Cox regressions for investigating the association between ACO and all-cause mortality, using non-ACO as reference. After adjusting for all potential confounders that represented demographic characteristics, health-related lifestyle and other medical conditions, and controlling for depressive disorders (Model 4 in Table 2), we found that referencing to participants without ACO, participants with ACO had a higher risk of all-cause mortality (HR = 1.856, 95%CI 1.535–2.245, *p* < 0.001). The R-squared, representing the proportion of the variance in the dependent variable that can be explained by the independent variables in the model, was 20.5% in Model 4. From the crude model to Model 4, by introducing the groups of covariables (demographic characteristics, health-related lifestyle and other medical conditions) gradually, R-square increased significantly, as all the values of F change were statistically significant. This suggested that the additional variables had a significant impact on the dependent variable, i.e., all-cause mortality, and the model’s explanatory power increased significantly as the focused controlled variables were added. In addition, across the models, the *p*-values for the trend analyses (*p* for trend) were all less than 0.001.

**Table 2:** Regressions exploring the association between ACO and all-cause mortality (comparing to non-ACO)

	**Crude model**			**Model 1**			**Model 2**			**Model 3**			**Model 4**		
	HR	95%CI	*P*	HR	95%CI	*P*	HR	95%CI	*P*	HR	95%CI	*P*	HR	95%CI	*P*
**Lung diseases**															
Non-ACO		ref			ref			ref			ref			ref	
ACO	2.821	2.313, 3.441	< 0.001	2.227	1.831,2.709	< 0.001	1.991	1.655,2.394	< 0.001	1.908	1.578,2.307	< 0.001	1.856	1.535,2.245	< 0.001
**Age**				1.095	1.090,1.101	< 0.001	1.084	1.079,1.090	< 0.001	1.095	1.089,1.101	< 0.001	1.096	1.090,1.102	< 0.001
**Gender** Female (ref) vs Male				1.687	1.537,1.851	< 0.001	1.568	1.425,1.725	< 0.001	1.525	1.367,1.701	< 0.001	1.539	1.381,1.716	< 0.001
**Race**															
Non-Hispanic Black					ref			ref			ref			ref	
Non-Hispanic White				1.077	0.949,1.222	0.342	1.145	1.017,1.298	0.007	1.224	1.076,1.392	0.002	1.216	1.072,1.381	0.002
Mexican American				0.636	0.529,0.764	< 0.001	0.701	0.584,0.840	< 0.001	0.845	0.670,01.022	0.083	0.837	0.693,1.013	0.068
Other races				0.719	0.583,0.887	< 0.001	0.753	0.612,0.926	< 0.001	0.898	0.729,01.105	0.309	0.887	0.721,1.090	0.253
**Education**															
Below high school					ref			ref			ref			ref	
High school or equivalent				1.030	0.915,1.161	0.664	1.033	0.916,1.165	0.596	1.100	0.960,1.260	0.168	1.107	0.966,1.268	0.144
College or above				0.902	0.781,1.041	0.135	0.892	0.776,1.025	0.108	1.068	0.917,1.244	0.398	1.079	0.928,1.255	0.323
**Marital status**															
Married					ref			ref			ref			ref	
Never married				1.753	1.427,2.155	< 0.001	1.773	1.436,2.189	< 0.001	1.799	1.443,2.243	< 0.001	1.784	1.437,2.214	< 0.001
Widowed/Divorced/Separated				1.411	1.276,1.560	< 0.001	1.438	1.296,1.595	< 0.001	1.382	1.244,1.536	< 0.001	1.372	1.235,1.524	< 0.001
**PIR**				0.790	0.759,0.822	< 0.001	0.809	0.776,0.844	< 0.001	0.846	0.810, 0.883	< 0.001	0.853	0.818,0.889	< 0.001
**DM** No (ref) vs Yes							1.463	1.322,1.620	< 0.001	1.422	1.273,1.589	< 0.001	1.414	1.265,1.580	< 0.001
**Hypertension** No (ref) vs Yes							1.278	1.145,1.427	< 0.001	1.238	1.108,1.384	< 0.001	1.233	1.104,1.376	< 0.001
**Hyperlipidemia** No (ref) vs Yes							0.751	0.665,0.848	< 0.001	0.751	0.648,0.857	< 0.001	0.753	0.660,0.858	< 0.001
**CVD** No (ref) vs Yes							1.706	1.545,1.883	< 0.001	1.548	1.428,1.758	< 0.001	1.562	1.406,1.734	< 0.001
**Cancer** No (ref) vs Yes							1.290	1.151,1.446	< 0.001	1.276	1.132,1.439	< 0.001	1.273	1.128,1.437	< 0.001
**BMI**							0.995	0.988,1.003	< 0.001	1.000	0.992,1.008	0.984	0.999	0.991,1.008	0.903
**HEI**										0.994	0.991,0.998	0.002	0.995	0.991,0.998	0.003
**Alcohol drinks ≥ 12 per year** No (ref) vs Yes										0.746	0.674,0.827	< 0.001	0.746	0.673,0.827	< 0.001
**Serum cotinine**										1.001	1.001,1.002	< 0.001	1.001	1.158,1.629	< 0.001
**Depressive disorders**^a^ No (ref) vs Yes													1.374	1.158,1.629	< 0.001
*P* for trend		< 0.001			< 0.001			< 0.001			< 0.001			< 0.001	
*R*-squared		0.007			0.181			0.193			0.204			0.205	
Adjusted *R*-squared		0.006			0.160			0.170			0.180			0.181	
F Change				3179.558, *p* < 0.001			141.072, *p* < 0.001			72.799, *p* < 0.001			12.395, *p* < 0.001		

Legends: Table 2 shows the results of weighted Cox regressions for investigating the association between ACO and all-cause mortality, using non-ACO as reference

Crude model: Non-adjusted

Model 1: Adjusted for age, gender, race, education, marital status, PIR

Model 2: Adjusted for age, gender, race, education, marital status, PIR, DM, hypertension, hyperlipidemia, CVD, cancer, BMI

Model 3: Adjusted for age, gender, race, education, marital status, PIR, DM, hypertension, hyperlipidemia, CVD, cancer, BMI, HEI, alcohol drink ≥ 12 per year, serum cotinine

Model 4: Adjusted for age, gender, race, education, marital status, PIR, DM, hypertension, hyperlipidemia, CVD, cancer, BMI, HEI, alcohol drink ≥ 12 per year, serum cotinine, depressive disorders

*Abbreviations*: *ACO* Asthma-chronic obstructive pulmonary disease overlap, *PIR* family poverty income ratio, *BMI* body mass index, *HEI* healthy eating index, *DM* diabetes mellitus, *CVD* cardiovascular disease, *CRD* clinically relevant depression*, HR* hazard ratio, *CI* confidence interval

^a^In this study those with PHQ-9 total scores ≥ 10 were considered as having depressive disorders or clinically relevant depression (CRD)

Table 3 shows the results of weighted cox regressions for investigating the association between ACO and all-cause mortality, with lung disease groups (ACO as the reference, asthma-only, COPD-only, and non-asthma/COPD) as the independent variable and all-cause mortality as the dependent variable. After adjusting for all potential confounders that represented demographic characteristics, health-related lifestyle and other medical conditions, and controlling for depressive disorders (Model 4 in Table 3), we found that referencing to participants with ACO, participants with other three conditions: asthma-only, COPD-only and non-asthma/COPD had lower mortality rates, with HRs = 0.411 (*p* < 0.001, 95%CI 0.311–0.543), 0.713 (*p* = 0.006, 95%CI 0.559–0.910) and 0.530 (*p* < 0.001, 95%CI 0.438–0.641) respectively. The R-squared of Model 4 was over 20%. Similar to Table 2, in Table 3, from the crude model to Model 4, by gradually adding the groups of controlled variables, R-square increased significantly, as all the values of F change were statistically significant. In addition, across the models, the *p*-values for the trend analyses (*p* for trend) were all less than 0.001.

**Table 3:** Regressions exploring the association between ACO and all-cause mortality (comparing to asthma-only, COPD-only, and non-asthma/COPD)

	**Crude model**			**Model 1**			**Model 2**			**Model 3**			**Model 4**		
	HR	95%CI	*P*	HR	95%CI	*P*	HR	95%CI	*P*	HR	95%CI	*P*	HR	95%CI	*P*
**Lung diseases**															
ACO		ref			ref			ref			ref			ref	
COPD-only	0.788	0.613,1.013	0.063	0.688	0.542,0.874	0.002	0.736	0.579,0.935	0.012	0.695	0.544,0.887	0.003	0.713	0.559,0.910	0.006
Asthma-only	0.252	0.195,0.327	< 0.001	0.376	0.293,0.484	< 0.001	0.403	0.314,0.518	< 0.001	0.404	0.307,0.532	< 0.001	0.411	0.311,0.543	< 0.001
Non-asthma/COPD	0.346	0.283,0.423	< 0.001	0.440	0.361,0.536	< 0.001	0.496	0.411,0.598	< 0.001	0.513	0.425,0.621	< 0.001	0.530	0.438,0.641	< 0.001
**Age**				1.095	1.089,1.100	< 0.001	1.084	1.078,1.089	< 0.001	1.092	1.086,1.098	< 0.001	1.093	1.088,1.099	< 0.001
**Gender** Female (ref) vs Male				1.646	1.495,1.813	< 0.001	1.531	1.386,1.691	< 0.001	1.530	1.371,1.707	< 0.001	1.544	1.385,1.721	< 0.001
**Race**															
Non-Hispanic Black					ref			ref			ref			ref	
Non-Hispanic White				1.056	0.930,1.200	0.518	1.126	0.990,1.280	0.07	1.197	1.048,1.367	0.008	1.189	1.044,1.355	0.009
Mexican American				0.633	0.526,0.762	< 0.001	0.697	0.581,0.836	< 0.001	0.831	0.685,1.009	0.062	0.720	0.597,0.869	0.05
Other races				0.709	0.574,0.877	< 0.001	0.745	0.604,0.920	< 0.001	0.874	0.708,1.081	0.214	0.863	0.700,1.065	0.170
**Education**															
Below high school					ref			ref			ref			ref	
High school or equivalent				1.023	0.909,1.152	0.740	1.023	0.907,1.155	0.707	1.096	0.958,1.255	0.183	1.102	0.963,1.262	0.159
College or above				0.901	0.781,1.040	0.132	0.891	0.775,1.025	0.107	1.052	0.903,1.226	0.516	1.064	0.915,1.237	0.423
**Marital**															
Married					ref			ref			ref			ref	
Never married				1.765	1.439,2.164	< 0.001	1.793	1.455,2.209	< 0.001	1.814	1.457,2.259	< 0.001	1.797	1.450,2.226	< 0.001
Widowed/Divorced/Separated				1.406	1.271,1.554	< 0.001	1.436	1.293,1.593	< 0.001	1.392	1.249,1.552	< 0.001	1.381	1.539,1.578	< 0.001
**PIR**				0.790	0.759,0.821	< 0.001	0.810	0.776,0.844	< 0.001	0.844	0.809,0.881	< 0.001	0.851	0.816,0.887	< 0.001
**DM** No (ref) vs Yes							1.464	1.318,1.626	< 0.001	1.415	1.264,1.584	< 0.001	1.408	1.258,1.577	< 0.001
**Hypertension** No (ref) vs Yes							1.257	1.122,1.408	< 0.001	1.257	1.122,1.408	< 0.001	1.251	1.118,1.400	< 0.001
**Hyperlipidemia** No (ref) vs Yes							0.752	0.667,0.848	< 0.001	0.747	0.656,0.851	< 0.001	0.749	0.658,0.852	< 0.001
**CVD** No (ref) vs Yes							1.792	1.530,1.871	< 0.001	1.581	1.424,1.756	< 0.001	1.556	1.400,1.730	< 0.001
**Cancer** No (ref) vs Yes							1.286	1.145,1.444	< 0.001	1.271	1.125,1.435	< 0.001	1.268	1.122,1.434	< 0.001
**BMI**							0.996	0.988,1.004	0.338	1.001	0.993,1.009	0.825	1.000	0.992,1.008	0.926
**HEI**										0.994	0.991,0.998	0.002	0.995	0.991,0.998	< 0.001
**Alcohol drinking ≥ 12 per year** No (ref) vs Yes										0.745	0.672,0.826	< 0.001	0.744	0.672,0.825	< 0.001
**Serum cotinine**										1.001	1.001,1.002	< 0.001	1.001	1.001,1.002	< 0.001
**Depressive disorders**^a^ No (ref) vs Yes													1.390	1.168,1.655	< 0.001
*P* for trend		< 0.001			< 0.001			< 0.001			< 0.001			< 0.001	
*R*-squared		0.014			0.184			0.196			0.204			0.205	
Adjusted *R*-squared		0.012			0.163			0.172			0.180			0.181	
F Change				3086.450, *p* < 0.001			134.212,* p* < 0.001			74.509, *p* < 0.001			12.731, *p* < 0.001		

Legends: Table 3 shows the results of weighted cox regressions for investigating the association between ACO and all-cause mortality, with lung disease groups (ACO as the reference, asthma-only, COPD-only, and non-asthma/COPD) as the independent variable and all-cause mortality as the dependent variable

Crude model: Non-adjusted

Model 1: Adjusted for age, gender, race, education, marital status, PIR

Model 2: Adjusted for age, gender, race, education, marital status, PIR, DM, hypertension, hyperlipidemia, CVD, cancer, BMI

Model 3: Adjusted for age, gender, race, education, marital status, PIR, DM, hypertension, hyperlipidemia, CVD, cancer, BMI, HEI, alcohol drink ≥ 12 per year, serum cotinine

Model 4: Adjusted for age, gender, race, education, marital status, PIR, DM, hypertension, hyperlipidemia, CVD, cancer, BMI, HEI, alcohol drink ≥ 12 per year, serum cotinine, depressive symptoms

*Abbreviations*: *ACO* Asthma-chronic obstructive pulmonary disease overlap, *COPD* Chronic Obstructive Pulmonary Disease, *PIR* family poverty income ratio, *BMI* body mass index, *HEI* healthy eating index, *DM* diabetes mellitus, *CVD* cardiovascular disease, *CRD* clinically relevant depression, *HR* hazard ratio, *CI* confidence interval

^a^In this study those with PHQ-9 total scores ≥ 10 were considered as depressive disorders or having clinically relevant depression (CRD)

### Mediating effect of depressive symptoms between ACO and all-cause mortality

As mentioned earlier, according to Table 1, participants in the ACO group were more likely to experience depressive disorders compared to those in the other groups. Tables 2 and 3 shows that after controlling for all covariates, ACO was significantly associated with all-cause mortality, and the presence of depressive disorders was found to increase all-cause mortality (*p* < 0.01). Table 4 presents the mediating effect of depressive symptoms in the association between ACO (non-ACO as the reference) and all-cause mortality, showing that after controlling for all covariates, the total effect, direct effect, and indirect effect of ACO on all-cause mortality were all significant (*p* < 0.001). The proportions mediated by depressive symptoms for ACO -associated all-cause mortality were 8.13% (CI: 4.22–14.00, *p* < 0.001).

**Table 4:** Mediating effect of depressive symptoms on the association between ACO and all-cause mortality

Independent variable	Mediator	Total effect		Indirect effect		Direct effect		Proportion mediated, % (95% CI)
		Coefficient (95% CI)	*P* value	Coefficient (95% CI)	*P* value	Coefficient (95% CI)	*P* value	
Asthma-chronic obstructive pulmonary disease overlap (ACO)	depressive symptoms	334.455(244.411,431.320)	< 0.001	27.66 (14.343,44.010)	< 0.001	306.794 (213.796,402.600)	< 0.001	8.13 (4.22,14.00)

The mediation analyses were adjusted for age, gender, race, education, marital, family poverty income ratio (PIR), diabetes mellitus (DM), hypertension, hyperlipidemia, cardiovascular disease (CVD), cancer, body mass index (BMI), healthy eating index (HEI), alcohol drinks ≥ 12 per year, and serum cotinine. The potential mediating effect of depressive symptoms on the association between ACO (non-ACO as reference) and all-cause mortality were estimated by using R package “mediation”. “survreg” method with 1000 simulations and 500 sims was applied. The total effect (TE) represented the influence of ACO on all-cause mortality without the hypothesized mediator, depressive symptoms. The direct effect (DE) represented the influence of ACO on all-cause mortality after controlling for the hypothesized mediator, depressive symptoms. The indirect effect (IE) represented the influence of ACO on all-cause mortality through the hypothesized mediator, depressive symptoms. The proportion of mediation was calculated by IE/TE

Legends: Table 4 presents the mediating effect of depressive symptoms in the association between ACO (non-ACO as the reference) and all-cause mortality, showing that after controlling for all covariates, the total effect, direct effect, and indirect effect of ACO on all-cause mortality were all significant (*p* < 0.001)

## Discussion

This study enhances current understandings of ACO by investigating its prevalence and epidemiological characteristics among a large population from the NHANES 2005–2018. The prevalence of ACO revealed by this study, 3.10%, was consistent with what was suggested in a review recently made by Alsayed et al. (2023) [2]. It reported that the prevalence of ACO in the general population ranged from 2 to 3 percent. Our finding was higher than what was observed by some previous studies based on NHANES, e.g., around 1% in Mendy et al. (2018) [26] with NHANES 2007–2012, 1.47% in Llanos et al. (2018) with NHANES 2009–2012 [1] with NHANES 2009–2012, and 2.88% in Wang et al. (2023) [4] with NHANES 1999–2018, which was probably due to the fact that our study included the data from the NHANES 2005–2018 that encompassed more recent years. This may suggest that the prevalence of ACO has increased in recent years. Our observation, the prevalence of ACO in the asthma patients was 23.08%, fell within the range of 10%- 31% suggested in Alsayed et al. (2023)’ review [2]. Our finding of the prevalence of ACO among COPD patients, which was 45.25%, aligned with the range of 4.2% to 66.0% in Leung and Sin (2022)’s suggestion [8]. Our study supported the current knowledge that ACO is not uncommon, especially among people with asthma or COPD.

Our study revealed some epidemiological characteristics of people with ACO. Our findings resonated Wang et al. (2023)’s [4] and Llanos et al. (2018)’s [1] discoveries with the NHANES database, showing that the mean age of participants with ACO was lower than that of those with COPD-only but higher than that of individuals with asthma-only. While in this study the ACO group had a more similar demographic pattern with the COPD-only group than the asthma-only group and the non-asthma/COPD group, the ACO group was significantly different from the COPD-only group in terms of gender, race and BMI, as the ACO group had more individuals from non-White population, more female, and more with a higher BMI, like what was suggested in Alsayed et al. (2023)’s [2] review. Similar to Wang et al. (2023) [4], this study noticed that individuals with ACO could be more likely to not only have a lower economic status but also a higher risk of suffering other diseases, like hypertension, DM, and CVD, compared to individual with asthma-only, COPD-only, or non-asthma/COPD. Thus, addressing comorbidity should be prioritized in managing ACO patients.

Furthermore, our study made an additional observation that individuals with ACO exhibited a higher likelihood of adopting an unhealthy dietary pattern reflected by a lower HEI. These results highlight the importance of providing dietary instructions to ACO patients and monitoring their dietary patterns. It is also noteworthy that our study revealed relatively lower alcohol consumption among individuals with ACO, which aligns with the findings reported by Wang et al. (2023) [4]. This observation suggests that individuals in the ACO group may be more inclined to prioritize their health and exercise control over their alcohol intake.

Aligning with the most population-based studies [4, 8, 62–67] that used different ACO definitions or identification criteria, our study suggests that individuals with ACO have a higher risk of all-cause mortality, compared to individuals from the groups of asthma-only, COPD-only, non-asthma/COPD, or non-ACO. However, our findings were notably different from conclusions given by many observational studies that comparing to patients suffering from other lung diseases, patients with ACO had similar or lower all-cause mortality risks [3, 68]. These contradictory conclusions have been noticed by Mekov et al. (2021) and Uchida et al. (2018) [3, 68], who suggested that these conflicting observations could be partially contributed to the better management delivered to the targeted ACO patients in observational studies. Our results, combined with the previous conclusions, imply that ACO may be associated with increased mortality risks and the implementation of appropriate management strategies should be important to mitigate the risks. Furthermore, it would be important to conduct more rigorously controlled research that accounts for various definitions and management approaches of ACO to obtain more reliable and confident insights into the ACO-associated all-cause mortality. In addition, it would be valuable to conduct comparative studies between different management approaches for ACO patients. This would further the understanding of how various treatment strategies influence health outcomes and help identify the most effective approaches to managing ACO.

While the relationship between mental health and other lung diseases (e.g., asthma and COPD) has been extensively explored [69], the incidence of depressive disorders among patients with ACO was little known [70]. Paying attention to the mental health of ACO patients, this study found that there was a higher proportion of people suffering from depressive disorders in the ACO group than in the asthma-only group, COPD-only group, non-asthma/COPD group, and non-ACO group. Using non-ACO participants as reference, these results revealed that ACO patients might have a higher risk of experiencing depressive disorders, which were consistent with the insights given by previous studies, e.g., Homętowska et al. (2022) [71] and Kang et al. (2019) [70], Chabowski et al. (2016) [72], and Lee et al. (2021) [15]. Although the present findings did not provide definitive evidence of the causal relationship between ACO and depressive states or conclusive reasons for the elevated risks of depression in ACO patients, several factors may contribute to this association. For instance, the increased severity of airflow obstruction, indicated by a lower FEV1/FVC ratio [73, 74], or the presence of more pronounced airway constriction and alveolar damage in ACO patients can progressively lead to respiratory failures, which in turn can result in cerebral hypoxia, carbon dioxide retention, and acidosis, causing damage to brain blood vessels and brain cells, and can impact the central nervous system, potentially amplifying negative emotions [75, 76]. The presence of an elevated risk of depressive disorders in the ACO group emphasizes the need for healthcare providers to pay attention to the mental well-being of ACO patients.

Our suggestion to address depressive symptoms in individuals with ACO is further supported by the evidence showing that depressive symptoms played a mediating role in the association between ACO and all-cause mortality and the mediating effect was significant, accounting for 8.47% of the total effect of ACO on all-cause death. According to the existed evidence presented above, ACO events may increase depressive symptoms, and people suffering from depression may have unhealthier lifestyles and self-harm behaviours that have consistently been shown to be causal risk factors of premature death [19]. Thus, giving careful attention to and effectively managing depressive symptoms may be beneficial to reduce all-cause mortality for those with ACO. By recognizing the increased risk of depressive disorders in ACO patients and the strong mediating role played by depressive symptoms in the development of ACO to death, healthcare professionals should deliver appropriate strategies to support their ACO patients. Necessary measures may include screening for and addressing depressive disorders, providing patient with the access to mental health resources, and integrating mental health care into the overall management of ACO. Additional research is necessary to determine whether the prevention and treatment of depressive symptoms can potentially enhance overall survival in individuals with ACO. Although there is little empirical evidence regarding the impact of addressing depressive symptoms on survival rates in ACO patients, managing depressive symptoms is still valuable for other reasons, such as potentially alleviating symptoms and improving the quality of life [77].

One noteworthy observation that requires special attention is the association between hyperlipidemia and the risk of all-cause mortality in our study population. Interestingly, our findings align with previous studies such as Yeramaneni et al. (2017) [78], which also reported a lower risk of all-cause mortality in individuals with hyperlipidemia. However, it is important to note that our results conflict with the conclusions drawn by other studies like Feng et al. (2022) [79]. Given the conflicting findings, it is crucial to conduct further studies to generate more robust evidence regarding the relationship between hyperlipidemia and all-cause mortality.

### Strengths and limitations

With a large population from the NHANES 2005–2018, our research advanced the current epidemiological understandings of ACO, particularly in relation to its psychological characteristics. To increase the robustness of our findings, we carefully considered and controlled for potential confounding factors, such as demographic factors, clinical factors and lifestyle factors, which were adjusted for in our regression analyses. One of the key strengths of our research is that it was the first study to investigate the underlying mechanisms linking ACO to mortality outcomes. Specifically, we explored the mediating role of depressive symptoms in the relationship between ACO and all-cause mortality. By examining the psychological pathway through which ACO may increase mortality, our study could help to fill the gap in the literature regarding the psychological characteristics of ACO and the role of mental health in ACO development. Moreover, aligning our approaches to identifying diseases and clinical conditions with that of other studies enhances the comparability of our findings with other relevant studies based on the NHANES dataset. Overall, this study could contribute to the existing knowledge that may inform clinical practice and interventions aimed at improving the health outcomes of ACO patients.

However, it is important to acknowledge the limitations of our study. Firstly, our study population was limited to individuals aged 40 and above, which may restrict the generalizability of our findings to younger age groups. Further research is needed to investigate the incidence trends in different age groups. Secondly, the scope of our study and the data availability may have constrained the selection and categorization of covariates, which might lead to the omission of certain important comorbidities that should be considered. More potential confounders should be included and addressed in future studies. Additionally, while our study investigated the mediating role of depressive symptoms in the association between ACO and all-cause mortality, it is important to acknowledge that there may be other mechanisms at play. Further research is needed to explore these additional mechanisms in order to gain a deeper understanding of the complex relationship between ACO and the mortality outcome. Another point that requires attention is the use of the PHQ-9, a commonly used tool for assessing depressive symptoms, rather than a diagnostic tool. It is important to note that the PHQ-9 scores have been found to be significantly associated with COPD and other comorbidities [69]. Given the present analyses, we cannot guarantee the complete removal of the effect of COPD and other comorbidities on PHQ-9 scores when assessing the association between ACO and depressive symptoms. Therefore, caution is advised when interpreting the mediating effect of depressive symptoms in the association between ACO and mortality as observed in this study.

It is also important to note that certain limitations within the NHANES database may introduce potential biases into this study. Primarily, considering the constraints of the data availability and the reliance on self-reported data within the NHANES, coupled with the absence of precise diagnostic information, it is essential to recognize the potential of making biases in the identification of those diseases or medical conditions considered in this study. Further, the NHANES database lacks the exact index date, which signifies the specific time when a diagnosis, examination or measurement is made. In our study, the absence of the information about the timing of diagnosis for lung diseases such as asthma and COPD hinders our ability to ascertain the lead time of these conditions at the point of an individual's recruitment into the survey. This limitation could potentially introduce biases when comparing the characteristics of different diseases, such as ACO versus asthma-only or COPD-only. In addition, the depressive symptoms of our participants were assessed at the time of their entry into the survey, and we are unable to establish the temporal sequence between depression and ACO or other lung diseases. As a result, this study can only exploratively investigate the association between lung health issues and depressive symptoms and statistically assess the potential role of depression in this association. Moreover, there was a lack of information of whether our participants had received any psychological supports or not. Thus, prospective longitudinal studies that track patients over time and control for any interventions are needed to establish the temporal sequence between lung diseases and depression more accurately and provide more robust evidence. Furthermore, this study used the time of survey participation as the baseline for constructing follow-up periods in time-to-event analyses, which may introduce certain biases to the results. All these limitations emphasize the need for caution in interpreting the results of the study and call for more rigorous longitudinal studies with follow-up data available between measurements, the incidence of diseases, and mortality.

## Conclusions

Using the NHANES 2005–2018, this cohort study indicates that ACO is not uncommon, especially among patients with asthma or COPD, and that individuals with ACO tend to have a lower socio-economic status, poorer health conditions, unhealthier dietary pattern, and decreased mental health status. In addition, this study sheds light on the enhanced risks of all-cause mortality among ACO patients and uncovers a potential psychological mechanism underlying the relationship between ACO and all-cause mortality. This study has important implications for clinical practice and public health management to improve the outcomes and quality of life for individuals living with ACO, suggesting the potential necessity of providing early detection, healthy lifestyle instruction, comprehensive comorbidity management, and mental health care to ACO patients, especially those living with disadvantaged conditions.

### Supplementary Information

Additional file 1. Proportional hazards assumption tests

## Data Availability

The National Health and Nutrition Examination Survey (NHANES) data are publicly available at https://www.cdc.gov/nchs/nhanes/index.htm.

## Abbreviations

95%CI: 95% Confidence interval
ACO: Asthma-chronic obstructive pulmonary disease overlap
BMI: Body mass index
CVD: Cardiovascular Disease
COPD: Chronic Obstructive Pulmonary Disease
CRD: Clinically relevant depression
DM: Diabetes mellitus
PIR: Family poverty income ratio
FEV1/ FVC: Forced expiratory volume/forced vital capacity
HR: Hazard ratio
HEI: Healthy eating index
HEI: Healthy eating index
NCHS: National Center for Health Statistics
NDI: National death index
NHANES: National Health and Nutrition Examination Survey
PHQ-9: Patient Health Questionnaire-9
MEC: The Mobile Examination Centre
