# Supplementary figures and images for: Epidemiological characteristics of asthma-COPD overlap, its association with all-cause mortality, and the mediating role of depressive symptoms: evidence from NHANES 2005–2018
Source: BMC Public Health. 2024 May 28;24:1423. doi: 10.1186/s12889-024-18911-1 (PMC11134654; doi:10.1186/s12889-024-18911-1)

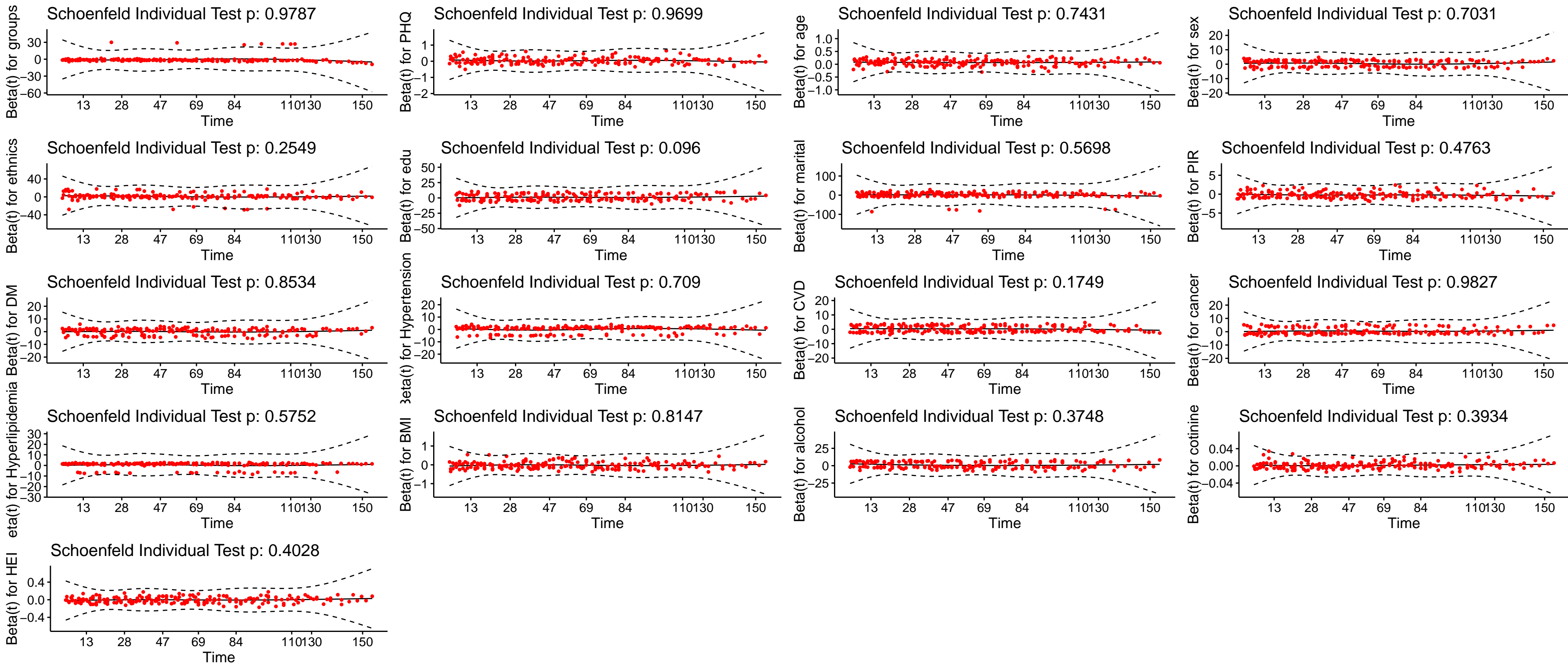

Supplement: Supplementary file 1 — Additional file 1. Proportional hazards assumption tests [file 12889_2024_18911_MOESM1_ESM.pdf]
